# Late biliary endoclip migration after laparoscopic cholecystectomy: Case report and literature review

**DOI:** 10.1016/j.ijscr.2020.08.027

**Published:** 2020-08-29

**Authors:** Hytham K.S. Hamid, Anna Fullard, Jamaleldin Sabahi, Sean M. Johnston

**Affiliations:** a Department of Surgery, Soba University Hospital, Khartoum, Sudan; b Department of Surgery, Midland Regional Hospital, Tullamore, Ireland

**Keywords:** Laparoscopic, Cholecystectomy, Endoclip, Migration

## Abstract

**Introduction:**

Although formerly considered a rarity, biliary endoclip-related complications are being diagnosed with increasing frequencies. Among these, migration of endoclip into the common bile duct (CBD) is a rare encounter that usually presents in the first two years after surgery.

**Presentation of case:**

This case demonstrates a late biliary endoclip migration after laparoscopic cholecystectomy.

**Discussion:**

An 82-year-old male patient, with a history of laparoscopic cholecystectomy 22 years ago, presented with two-day history of severe upper abdominal pain. Routine hemogram and serum chemistry were remarkable for slightly raised alanine aminotransferase and C-reactive protein. A computed tomographic (CT) scan demonstrated a new metallic density within the CBD when compared to a previous CT scan 14 months earlier. An endoscopic retrograde cholangiography confirmed a metal endoclip within the mid-CBD contained within a choledochal stone. Balloon extraction of the endoclip and stone was successfully performed. The patient was discharged 2 days later, and remained symptom free for 1 year.

**Conclusion:**

To our knowledge, a latency of 22 years between cholecystectomy and clip migration has never been reported before. In cases of post-cholecystectomy abdominal pain the awareness of the surgeon should always be drawn to a clinical suspicion of endoclip migration into the CBD that can be easily remedied. Endoscopic biliary sphincterotomy with endoclip/stone removal is the therapeutic procedure of choice which usually circumvents the need for surgical extraction.

## Highlights

• Biliary endoclip migration (ECM) may present as many as 22 years after laparoscopic cholecystectomy.
• Diagnosis of ECM should always be considered after cholecystectomy in patients presenting with abdominal pain.
• Increased attentiveness to the placement location and judicious limitation of the use of endoclips is advised.

## 1 Introduction

Laparoscopic cholecystectomy (LC) remains an accepted and widely used treatment modality for symptomatic cholelithiasis. Endoclips are often used during LC to achieve reliable and efficient haemostasis and to control the biliary tree terminals. Migration of these endoclips is uncommon and is clinically silent in the majority of patients [1]. However, at times, serious complications may arise due to endoclip burrowing into adjacent structures [2]. We present an interesting case of late migration of a biliary endoclip in an elderly patient, which ultimately caused biliary obstruction. The clinical course, radiological findings and management are discussed with review of the pertinent literature. This case was handled at an academic institution and has been presented in accordance with the SCARE criteria [3].

## 2 Case report

**Fig. 1:**
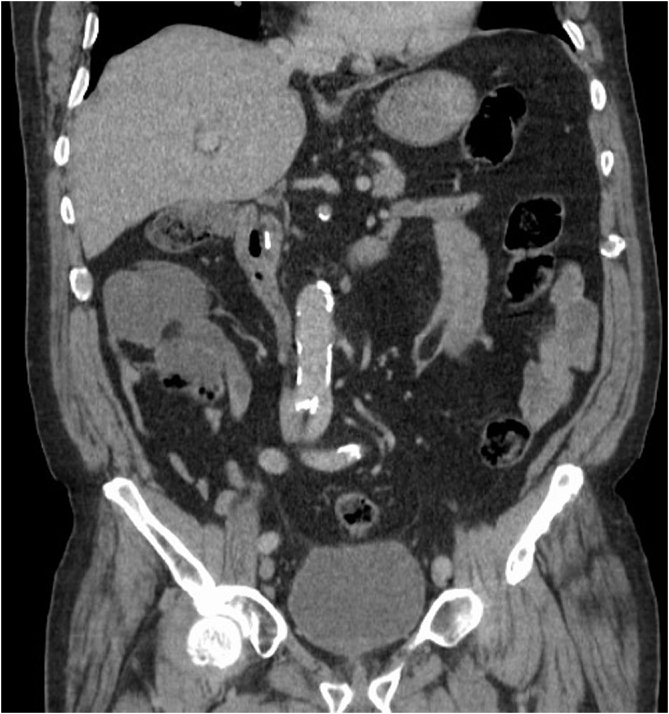
Coronal enhanced CT scan exhibiting a linear metallic density in the CBD consistent with a surgical endoclip.

**Fig. 2:**
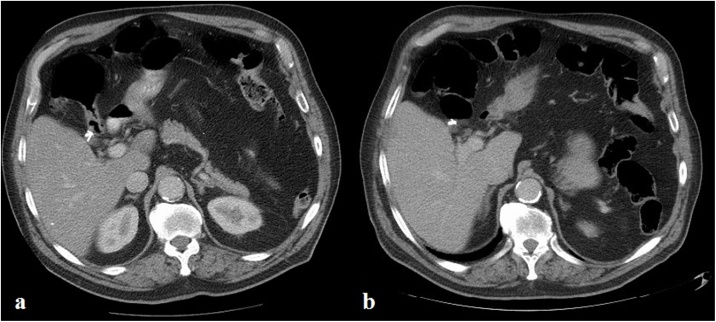
**a**, **b** Axial enhanced CT scan demonstrating three endoclips in the hepatic hilum (a) compared with two endoclips in the same location 14 months later (b).

An 82-year-old male patient presented with two-day history of nausea and severe upper abdominal pain, which was deteriorating over several hours. Despite opioid analgesia, his pain was not adequately controlled. He denied any other symptoms pertaining to intestinal or urological organ systems. His medical history was notable for appendicectomy, ischemic heart disease, and hypertension for which he was on anti-hypertensive medications. The patient also had uneventful interval laparoscopic cholecystectomy 22 years earlier, which was performed by a surgeon who was in his learning curve for laparoscopy at the time. During the procedure, three endoclips were used to ligate the cystic duct. He was living with his family and there was no family history of similar condition. Abdominal examination showed severe epigastric and right hypochondrial tenderness. Laboratory tests disclosed a mildly raised alanine aminotransferase (ALT) of 115 U/L (N: 8–41 U/L) and CRP of 15.6 mg/L (N: 0–5 mg/L), but otherwise were normal. A computed tomographic (CT) scan of the abdomen demonstrated a moderately dilated CBD (9 mm), containing a new metallic density when compared to a previous CT scan 14 months earlier (Fig. 1), whereas the number of surgical endoclips at the hepatic hilum has decreased from three to two (Fig. 2**a, b**). A clinical diagnosis of endoclip migration into the CBD was entertained.

**Fig. 3:**
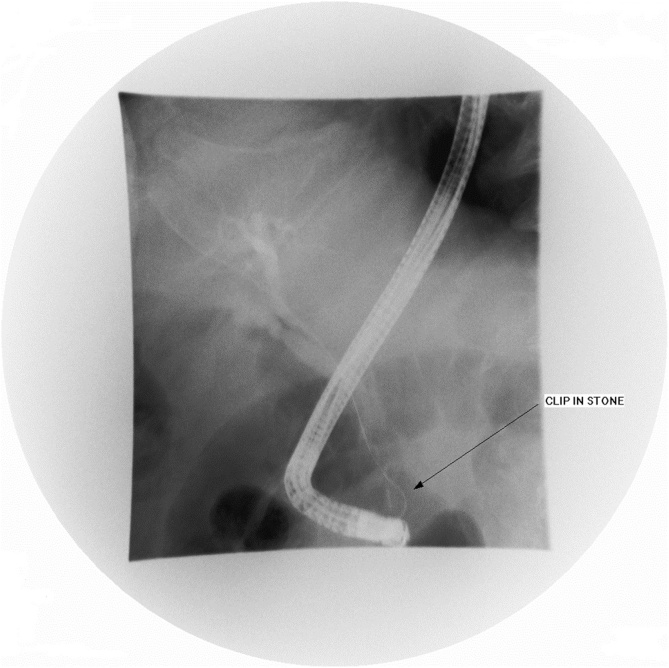
ERCP showing the clip/stone adjacent to the head of the endoscope in the duodenum, following successful extraction.

**Fig. 4:**
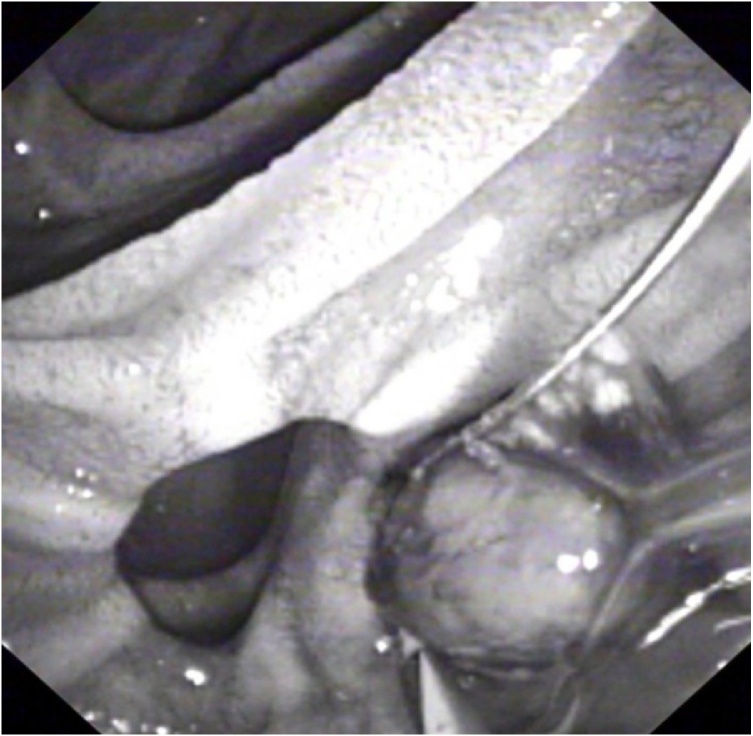
ERCP showing the stone in the duodenum after extraction.

Three days following admission, the patient developed cholestatic jaundice and fever. Liver biochemistry showed the following values: total bilirubin 48 gm/dl (N: <21 gm/dl), gamma-glutamyl transferase 771 U/L (N: 8–61 U/L), alkaline phosphatase 140 IU/L (N: 40–129 IU/L), aspartate aminotransferase 175 IU/L (N: 10–40 IU/L), and ALT 182 U/L (N: 8–41 U/L). The patient was transferred to a specialized endoscopy centre. Urgent ERCP revealed a solitary mid-CBD filling defect encasing a linear metallic radiodensity consistent with a gallstone and embedded endoclip (Fig. 3). Medium-sized sphincterotomy was successfully performed, and the stone and clip were extracted using a retrieval balloon catheter (Extractor™ Pro RX, Boston Scientific, Cork, Ireland) and released into the duodenum (Fig. 4). Gross examination of the stone revealed a closed endoclip.

Although the patient tolerated the endoscopic procedure well and was satisfied with the outcome, his recovery was complicated by atrial fibrillation requiring medical treatment. He was discharged home 2 days later with normal amylase and bilirubin, and he was symptom free 1 year later.

## 3 Discussion

Endoclip migration (ECM) following LC was first described by Onghena et al. in 1992 [4]. This was further elucidated by Martinez and colleagues, who reported two cases of choledocholithiasis secondary to ECM, which were successfully treated endoscopically [5]. According to previous reports, the estimated incidence of ECM in clinical materials ranges between 1% and 15%, depending on the level of cystic duct dissection performed and the number of clips used to attain haemostasis and biliostasis [1]. Most displaced endoclips migrate along the tissue planes with no major consequences. In rare instances, the endoclips may erode into adjacent structures such as the duodenum and the extrahepatic biliary tract, and promotes lithogenesis. Penetration of the ductal system usually manifests in the first two postoperative years as recurrent abdominal pain, whilst obstructive jaundice and cholangitis are the main modes of presentation in patients with concomitant choledocholithiasis [2,6,7]. Other complications include choledochoduodenal fistula, hepatolithiasis, pancreatitis, bile leak, and biliary strictures [[8], [9], [10], [11], [12], [13]].

Several hypotheses have been advanced for the mechanisms involved in ECM. The presence of acute cholecystitis, postoperative infection or bile leak in some cases points to acute local inflammation and direct cystic duct remnant or CBD erosion as possible underlying mechanisms [2]. These may explain ECM in patients presenting early during the postoperative course. Other important factors related to technical considerations include inaccurate endoclip placements, inadvertent bile duct injuries, and short cystic duct stump. Additionally, a prospective study has evidenced a significantly increased propensity for ECM in patients who had more than four endoclips applied during LC [1]. In cases with late ECM, however, the pathogenesis remains enigmatic. Although endoclips are made of relatively inert materials, they do cause a certain degree of host reaction [14]. We postulate that a low-grade foreign body inflammatory process induced by the endoclips, which often become redundant, contributes to late ECM and eventually burrowing into adjacent structures.

Diagnosis of ECM requires high index of clinical suspicion and is frequently made at the radiological level. Serial abdominal radiographs and cross-sectional imaging can prove helpful in detecting early displacement of endoclips, and the term “cat’s eye calculus” is often used to denote the endoclip-stone appearance on CT scan [15]. ERCP remains the gold standard to confirm the diagnosis and along with sphincterotomy offers the best treatment modality with success rate of 85% [2]. Both balloon catheter or wire basket can be safely used for endoclip-stone retrieval. Surgical exploration and percutaneous transhepatic cholangiography should be reserved as rescue procedures for cases in which endoscopic therapy is either not feasible or fails [16]. The latter could be due to the unfavourable endoclip-stone orientation within the CBD or the presence of biliary strictures, fistulas, or large stones [2,17].

## 4 Conclusion

ECM is a rare occurrence that may result in potentially life-threatening complications. To our knowledge, this is the first case reported in the literature of ECM with a latency of over 20 years between cholecystectomy and clinical presentation. Regardless of the time interval from surgery, the diagnosis of ECM should always be considered following LC. A possible association between the incidence of ECM and the number and position of deployed endoclips prompts increased attentiveness to the placement location and judicious limitation of the use of endoclips during surgery. The use of absorbable endoclips and ligatures in LC may play a role in preventing endoclip-related complications, and may herald a future with a curtailed role for the traditional titanium clips.

## Declaration of Competing Interest

None declared.

## Funding

None declared.

## Ethical approval

Not required.

## Consent

Written informed consent was obtained from the patient’s wife for publication of this case report and accompanying images. A copy of the written consent is provided for review by the Editor-in-Chief of this journal. The patient is not fit to give a consent.

## Author contribution

Study Concept and Design: H. Hamid, J. Sabahi, S. Johnston.

Data Collection: H. Hamid, J. Sabahi, A. Fullard.

Writing of Paper: H. Hamid, A. Fullard.

Critical revision for intellectual contents: S. Johnston.

## Registration of research studies

NA.

## Guarantor

Hytham K. S. Hamid.

## Provenance and peer review

Not commissioned, externally peer-reviewed.

## References

[1] Cetta F., Baldi C., Lombardo F., Monti L., Stefani P., Nuzzo G. (1997). Migration of metallic clips used during laparoscopic cholecystectomy and formation of gallstones around them: surgical implications from a prospective study. J. Laparoendosc. Adv. Surg. Tech. A.

[2] Chong V.H., Chong C.F. (2010). Biliary complications secondary to post-cholecystectomy clip migration: a review of 69 cases. J. Gastrointest. Surg..

[3] Agha R.A., Borrelli M.R., Farwana R., Koshy K., Fowler A., Orgill D.P., for the SCARE Group (2018). The SCARE 2018 statement: updating consensus surgical CAse REport (SCARE) guidelines. Int. J. Surg..

[4] Onghena T., Vereecken L., Van den Dwey K., Van Loon C. (1992). Common bile duct foreign body; an unusual case. Surg. Laparosc. Endosc..

[5] Martinez J., Combs W., Brady P.G. (1995). Surgical clips as a nidus for biliary stone formation: diagnosis and therapy. Am. J. Gastroenterol..

[6] Ray S., Bhattacharya S.P. (2013). Endoclip migration into the common bile duct with stone formation: a rare complication after laparoscopic cholecystectomy. JSLS.

[7] Sormaz I.C., Keskin M., Sönmez R.E., Soytaş Y., Tekant Y., Avtan L. (2015). Obstructive jaundice secondary to endoclip migration into common bile duct after laparoscopic cholecystectomy. Minerva Chir..

[8] Sato T., Denno R., Yayama Y., Matsuura T., Kanisawa Y., Hirata K. (1994). Unusual complications caused by endo-clip migration following a laparoscopic cholecystectomy: report of a case. Surg. Today.

[9] Mouzas I.A., Petrakis I., Vardas E., Kogerakis N., Skordilis P., Prassopoulos P. (2005). Bile leakage presenting as acute abdomen due to a stone created around a migrated surgical clip. Med. Sci. Monit..

[10] Dolay K., Alis H., Soylu A., Altaca G., Aygun E. (2007). Migrated endoclip and stone formation after cholecystectomy: a new danger of acute pancreatitis. World J. Gastroenterol..

[11] Torres O.J.M., Neiva R.F., Torres C.C.S., Freitas T.M., Fernandes E.S.M. (2018). Right hepatectomy due to hepatolithiasis caused by endoclip migration after laparoscopic cholecystectomy: a case report. J. Surg. Case Rep..

[12] Hong T., Xu X.Q., He X.D., Qu Q., Li B.L., Zheng C.J. (2014). Choledochoduodenal fistula caused by migration of endoclip after laparoscopic cholecystectomy. World J. Gastroenterol..

[13] Çolak Ş., Bektaş H., Gürbulak B. (2020). Mechanical jaundice and bile duct stricture caused by the migration of endoclip and silk suture material into the common bile duct. J. Coll. Physicians Surg..

[14] Mateo R., Tsai S., Stapfer M.V., Sher L.S., Selby R., Genyk Y.S. (2008). Ischemic mass effect from biliary surgical clips. J. Laparoendosc. Adv. Surg. Tech. A.

[15] Wu W.C., Katon R.M., McAfee J.H. (1993). Endoscopic management of common bile duct stones resulting from metallic surgical clips (cat’s eye calculi). Gastrointest. Endosc..

[16] Williams E.J., Green J., Beckingham I., Parks R., Martin D., Lombard M., British Society of Gastroenterology (2008). Guidelines on the management of common bile duct stones (CBDS). Gut.

[17] Tsumura H., Ichikawa T., Kagawa T., Nishihara M., Yoshikawa K., Yamamoto G. (2002). Failure of endoscopic removal of common bile duct stones due to endoclip migration following laparoscopic cholecystectomy. J. Hepatobiliary. Surg..

